# PVP-coated, negatively charged silver nanoparticles: A multi-center study of their physicochemical characteristics, cell culture and in vivo experiments

**DOI:** 10.3762/bjnano.5.205

**Published:** 2014-11-03

**Authors:** Sebastian Ahlberg, Alexandra Antonopulos, Jörg Diendorf, Ralf Dringen, Matthias Epple, Rebekka Flöck, Wolfgang Goedecke, Christina Graf, Nadine Haberl, Jens Helmlinger, Fabian Herzog, Frederike Heuer, Stephanie Hirn, Christian Johannes, Stefanie Kittler, Manfred Köller, Katrin Korn, Wolfgang G Kreyling, Fritz Krombach, Jürgen Lademann, Kateryna Loza, Eva M Luther, Marcelina Malissek, Martina C Meinke, Daniel Nordmeyer, Anne Pailliart, Jörg Raabe, Fiorenza Rancan, Barbara Rothen-Rutishauser, Eckart Rühl, Carsten Schleh, Andreas Seibel, Christina Sengstock, Lennart Treuel, Annika Vogt, Katrin Weber, Reinhard Zellner

**Affiliations:** 1 See end of main text.

**Keywords:** aerosols, biological properties, cell biology, nanoparticles, nanotoxicology, silver

## Abstract

PVP-capped silver nanoparticles with a diameter of the metallic core of 70 nm, a hydrodynamic diameter of 120 nm and a zeta potential of −20 mV were prepared and investigated with regard to their biological activity. This review summarizes the physicochemical properties (dissolution, protein adsorption, dispersability) of these nanoparticles and the cellular consequences of the exposure of a broad range of biological test systems to this defined type of silver nanoparticles. Silver nanoparticles dissolve in water in the presence of oxygen. In addition, in biological media (i.e., in the presence of proteins) the surface of silver nanoparticles is rapidly coated by a protein corona that influences their physicochemical and biological properties including cellular uptake. Silver nanoparticles are taken up by cell-type specific endocytosis pathways as demonstrated for hMSC, primary T-cells, primary monocytes, and astrocytes. A visualization of particles inside cells is possible by X-ray microscopy, fluorescence microscopy, and combined FIB/SEM analysis. By staining organelles, their localization inside the cell can be additionally determined. While primary brain astrocytes are shown to be fairly tolerant toward silver nanoparticles, silver nanoparticles induce the formation of DNA double-strand-breaks (DSB) and lead to chromosomal aberrations and sister-chromatid exchanges in Chinese hamster fibroblast cell lines (CHO9, K1, V79B). An exposure of rats to silver nanoparticles in vivo induced a moderate pulmonary toxicity, however, only at rather high concentrations. The same was found in precision-cut lung slices of rats in which silver nanoparticles remained mainly at the tissue surface. In a human 3D triple-cell culture model consisting of three cell types (alveolar epithelial cells, macrophages, and dendritic cells), adverse effects were also only found at high silver concentrations. The silver ions that are released from silver nanoparticles may be harmful to skin with disrupted barrier (e.g., wounds) and induce oxidative stress in skin cells (HaCaT). In conclusion, the data obtained on the effects of this well-defined type of silver nanoparticles on various biological systems clearly demonstrate that cell-type specific properties as well as experimental conditions determine the biocompatibility of and the cellular responses to an exposure with silver nanoparticles.

## Review

### Introduction

Silver in the form of ions and nanoparticles is extensively used in consumer products and medical devices [1–11]. This is due to its well-known antibacterial action. However, there are increasing concerns about potential risks to humans and to the environment, especially in the case of silver nanoparticles [12–19]. The assessment of the physicochemical and biological properties of silver nanoparticles is complicated because these properties depend on a number of parameters, such as size, shape, charge, dispersion state, and surface functionality. Therefore, the comparison of the results from different groups is typically difficult because either different particles were used or different chemical or biological methods were applied (see [1,20] for literature surveys). We have therefore performed a multi-center study in which the same kind of silver nanoparticles was applied by different groups. It is expected, therefore, that the obtained results have a high degree of comparability.

The silver nanoparticles were chemically characterized, purified from synthesis by-products and the silver content in the dispersions was measured for each batch. Unless otherwise noted, in all cases in which silver nanoparticles are referred to in the following sections, they are PVP-coated with a negative zeta potential of −20 mV and a metallic core diameter of about 70 nm. All concentrations given refer to the amount of silver.

This review article summarizes the results of all groups who participated in this study.

### Synthesis and colloid-chemical characterization of silver nanoparticles

**Figure 1:**
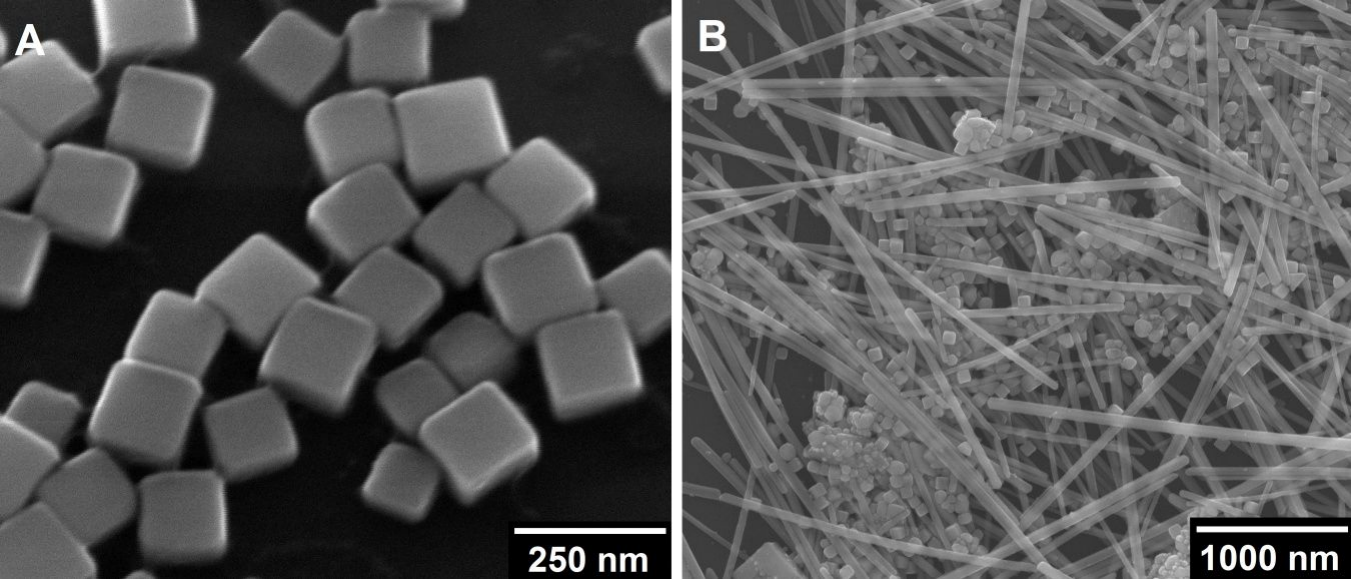
SEM images of silver nanocubes (A) and a mixture of silver nanoparticles with different shapes and sizes (B), both obtained from the polyol synthesis as described by Xia et al. [24] and carried out under exactly the same conditions.

The synthesis of silver nanoparticles with defined shapes and sizes is extensively described in the literature, with more than 50 publications alone by the group of Xia et al. [21]. The most common and best examined method is the polyol process during which an ionic silver salt (typically silver nitrate or silver trifluoroacetate) is reduced by the solvent ethylene glycol at temperatures of 140–180 °C in the presence of poly(*N*-vinylpyrrolidone); PVP) [22]. PVP serves as capping agent and stabilizes the formed nanoparticles against agglomeration, but also plays a role in the formation of specific shapes such as cubes or wires [23]. The purity of the products formed can be increased by the addition of trace amounts of HCl or NaHS that are believed to adsorb on specific crystal surfaces and thereby to control the crystal growth. They may also lead to the formation of sub-microscopic nuclei onto which silver nanoparticles sequentially grow [24–25]. Nevertheless, these syntheses are very sensitive towards almost every possible reaction parameter, e.g., temperature, concentration, but also the shape of the stirring bar or the manufacturer of the chemicals used [26–27] and therefore lack a reliable reproducibility. Figure 1 shows typical results of two identical syntheses.

Although it would be of interest to examine the influence of the shape on the biological impact of silver nanoparticles, it is crucial for biological experiments to choose a synthetic pathway that reliably produces particles of high quality and in large quantities over a large number of experiments. Because of this, we decided to synthesize our particles by the reduction of silver nitrate with glucose in the presence of PVP according to Wang et al. [28]. This leads to high yields of spherical silver nanoparticles with diameters of around 70–120 nm and a few triangular particles as byproduct [29].

**Figure 2:**
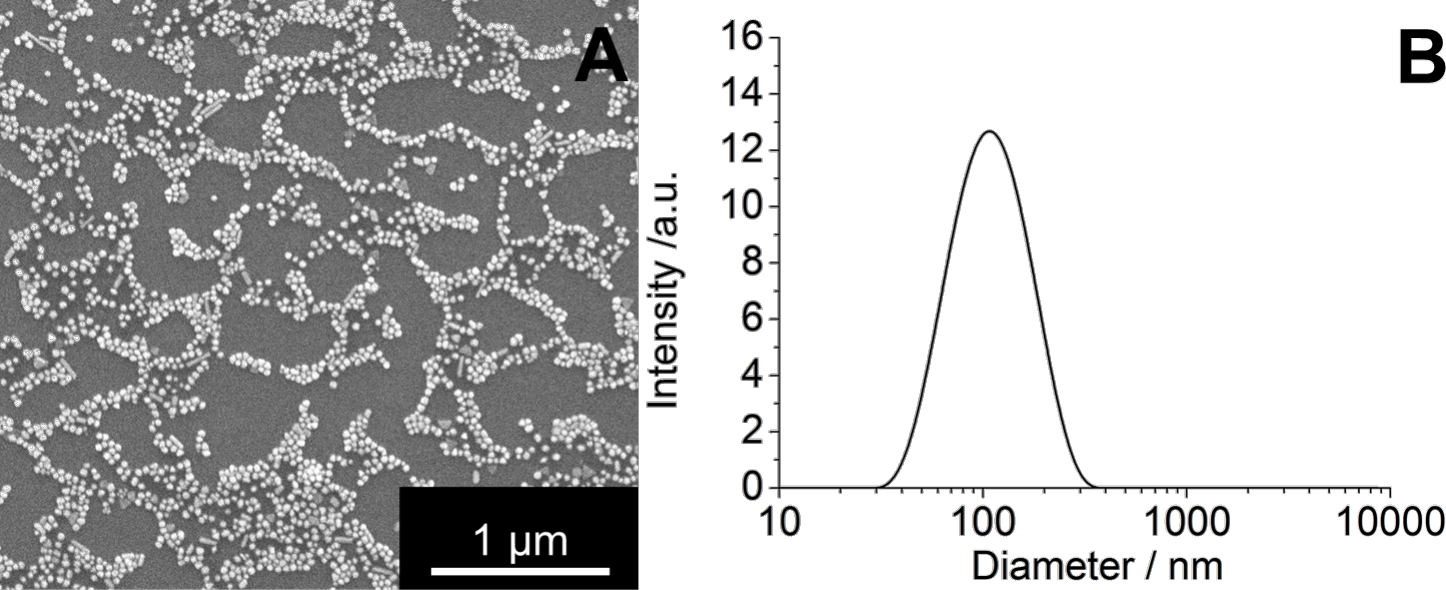
Representative scanning electron microscopy image of PVP-coated silver nanoparticles (A) and particle size distribution as measured by dynamic light scattering (B). These particles were used in all described experiments.

Figure 2A shows a typical SEM image of our silver nanoparticles. The diameter of the metallic core is about 70 nm. The hydrodynamic diameter as determined through dynamic light scattering is about 120 nm. The polydispersity index (PDI) was lower than 0.3 in all cases, which is indicative for a monodisperse system. The particles were negatively charged with a zetapotential of −20 mV. These particles were used in all described experiments after thorough chemical and colloid-chemical characterization.

### Dissolution of dispersed silver nanoparticles

Silver nanoparticles undergo dissolution in water due to oxidation by dissolved oxygen [30–33]. This leads to the release of silver ions, which are the toxic agent towards cells and bacteria [20,29,33–36]. The dissolution of silver nanoparticles in water and other media has been studied by a number of groups [20,30–33,37–41]. Typically, the dissolution is fast at the beginning of the experiment and slows down over time, leading to incompletely dissolved particles [33]. In the absence of oxygen, no dissolution occurs [20]. As a consequence, there is also no bactericidal effect in the absence of oxygen as shown by a very elegant experiment by Xiu et al. [34].

We extended our studies by the addition of a number of essential components of biological media, such as inorganic salts that can lead to the precipitation of sparingly soluble silver salts (AgCl and Ag_3_PO_4_), glucose as reducing sugar, and cysteine as a model compound for sulfur-containing proteins. H_2_O_2_ was used as strongly oxidizing compound. Furthermore, we analyzed the behavior of silver nanoparticles after their immersion in cell culture media (DMEM, RPMI and LB medium) [20].

**Figure 3:**
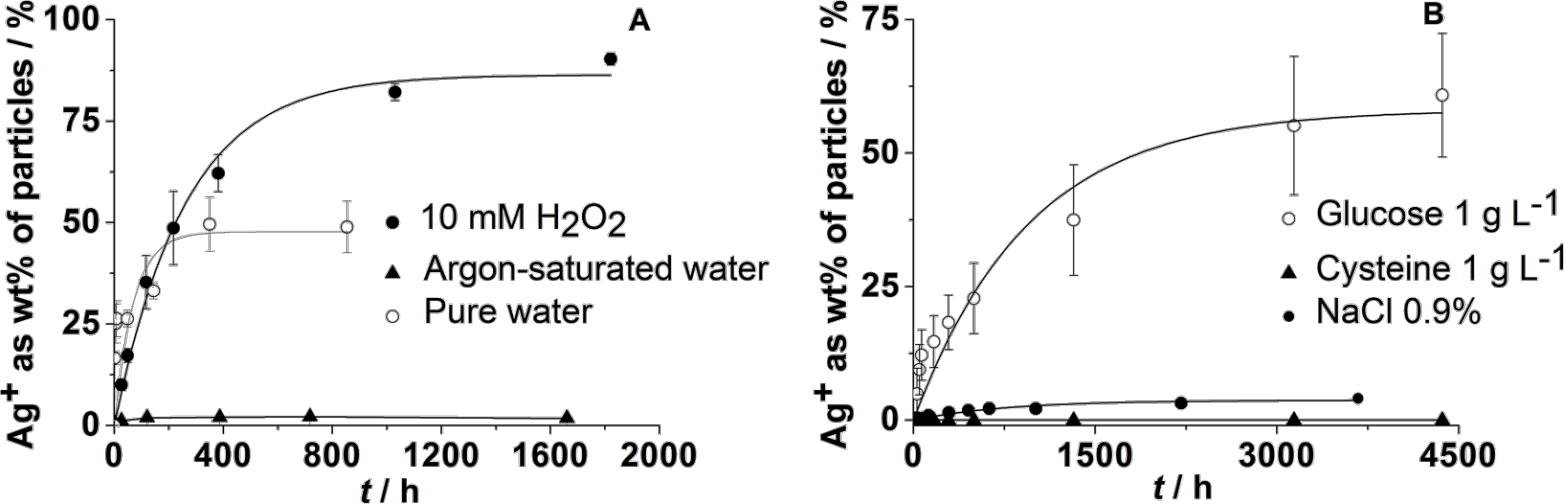
Dissolution of silver nanoparticles immersed in pure water, argon-saturated water under argon atmosphere, and in water with 10 mM H_2_O_2_ (A), in aqueous NaCl (0.9%), and in the presence of either cysteine (aq; 1 g L^−1^) or glucose (aq; 1 g L^−1^) (B). The data were taken from [20,33].

Figure 3A shows that the dissolution requires the presence of dissolved oxygen. If no oxygen is available, i.e., under argon atmosphere while using degassed water as solvent, only a very small fraction of silver is dissolved. This is probably due to traces of oxygen in the system. If a strongly oxidizing agent such as H_2_O_2_ is present, the dissolution rate is strongly increased. On the other hand, the dissolution is significantly slower in the presence of dissolved NaCl, a fact that may be due to the formation of insoluble silver chloride. An even stronger inhibiting effect is exerted by the sulfur-containing amino acid cysteine. We assume that this is due to a strong binding of the thiol group to the silver metal surface, which prevents the dissolution by passivation. Glucose, which is often used in syntheses to reduce silver ions to silver metal, has a decelerating effect but leads to a similar fraction of silver being finally dissolved (Figure 3B). This suggests that it either reduces some of the released silver ions or reduces the dissolved oxygen. Summarizing all these data, we have formulated a model for the oxidative dissolution of silver nanoparticles, based on the dissolution data of our group and of other groups. This model is discussed in detail in [20]. It mainly involves an oxidative dissolution of silver nanoparticles, typically by dissolved oxygen, and a passivation of the surface by chloride and sulfur-containing biomolecules.

So far, there are no quantitative data on the dissolution of silver nanoparticles in complex biological media. Considering the available literature data (see [1,12,20,31,42–43]), it can be assumed that silver ions are complexed by biomolecules and that silver nanoparticles are passivated in the presence of sulfide, sulfur-containing components and chloride. This passivation slows down the release of the toxic silver ions. The formation of nanoscopic silver chloride may also be responsible for the cytotoxicity of silver [44].

### The protein corona around silver nanoparticles

It is now well accepted that nanoparticles acquire a protein corona after contact with biological media [45–47]. This influences their dispersability in biological media, as we have shown for this particular kind of silver nanoparticles, and also their cytotoxicity [48]. This effect is, of course, not limited to silver nanoparticles [45,49–53]. The quantitative description of protein adsorption onto nanoparticle surfaces is a critical step towards understanding the formation of the protein corona at the full complexity of the physiological situation [45,54–58]. We have investigated the formation of a protein corona of serum albumin around silver nanoparticles [56]. Specifically, we have addressed the effect of a PVP coating around the metallic surface of silver and, for comparison, gold nanoparticles, on the adsorption/desorption equilibrium of serum albumin molecules – an established model protein [57,59–62].

To quantify this equilibrium, we have used circular dichroism (CD) spectroscopy in a quantitative approach allowing for the determination of equilibrium constants or binding affinities (transition midpoints) [55,59,63]. Circular dichroism signals of proteins arise from electronic transitions in specific secondary structural elements (e.g., α-helix or random coil), and we monitored them in dependence of the specific nanoparticle surface area as present in solution at a constant protein concentration. Making use of the fact that proteins can undergo substantial structural changes upon adsorption onto many nanoparticle surfaces [59,63–68], the quantitative analysis of the corresponding CD signals has been shown to be a good indicator for the overall extent to which the original secondary structure of serum albumin is preserved in the "equilibrated" corona [54,69–70]. The exact mechanisms of partial or full protein denaturation on such surfaces remain elusive, but first insights have been provided for insulin adsorption onto gold nanoshells [54].

**Figure 4:**
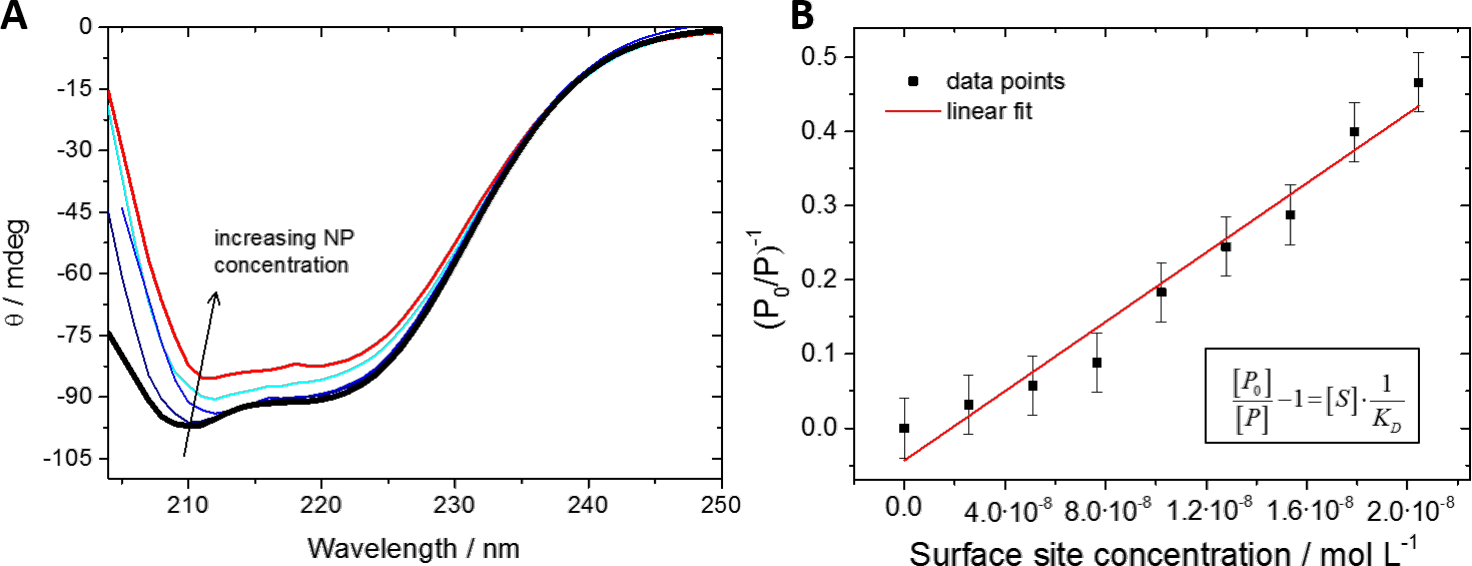
(**A**) CD spectra of pure dissolved bovine serum albumin (thick black line) and in the presence of different concentrations of PVP-coated silver nanoparticles. The concentration of the nanoparticles ranged from 1.20·10^11^ to 1.91·10^11^ nanoparticles mL^−1^. (**B**) Linear fit to the evaluated data for the determination of a *K*_D_ value for these nanoparticles according to the equation given in [63,69]. Note that the accuracy of the computed *K*_D_ value is determined by the accuracy of the CD spectra and the resulting values for the occupation of surface sites by the protein.

By expressing the equilibrium constant in terms of the number of surface sites, as determined by the sum over all particles multiplied by the maximum number of protein molecules that can fit on this surface, and the protein content in solution, we have derived an equation which links the experimental parameters of the CD measurements to the equilibrium constant, *K*_D_ [54,63,69]. Figure 4 shows typical CD spectra determined for pure serum albumin and the same protein solution containing PVP-coated silver nanoparticles at various concentrations as well as the corresponding analytical plot.

Equilibrium constants for the adsorption of albumin to silver and gold nanoparticles with and without a PVP coating were determined, revealing the influence of the polymer coating on the ability of the nanoparticles to adsorb proteins on their surface [69]. The results of these experiments are summarized in Table 1.

**Table 1:** Equilibrium constants *K*_D_ for PVP-coated silver nanoparticles and for silver nanoparticles stabilized by citrate ligands. Nanoparticle diameters were measured as indicated in the table, *K*_D_ values were determined from CD spectroscopic measurements [69]. For comparison, the same data are given for gold nanoparticles with the same surface functionalization.

nanoparticle	diameter [nm]	*K*_D_ [µM]

Ag (PVP; particles described in this article)	70 ± 6 (DLS)	0.5 ± 0.05
Au (PVP)	39 ± 3 (DLS)	0.2 ± 0.05
Ag (citrate)	40 ± 10 (SEM)	0.020 ± 0.0011
Au (citrate)	13 ± 2 (SEM)	0.033 ± 0.0032

For citrate-stabilized nanoparticles, we found affinities in the low nanomolar concentration regime, indicating a very high affinity of proteins to the nanoparticle surface. The affinities were one order of magnitude lower when PVP coatings were applied to the nanoparticles prior to protein adsorption, irrespective of the metallic core (silver or gold). This is a good indication of how PVP can shield the metallic surface of the nanoparticle and shows how the coating mediates the particle interaction with the environment.

The persistence of such a coating under physiological conditions emerges as an important aspect for understanding interactions between nanoparticles and biological entities in general [70]. Polymer coatings are frequently stable under such conditions but other ligands can well be replaced in equilibrium-type reactions even under chemically less complex conditions [54,71].

### Uptake of silver nanoparticles by various cell types

The exact fate of silver nanoparticles after uptake in cells is still unclear. This includes questions such as where they are located in cells and how they move within cells. Another question of interest concerns where the dissolution takes place. Are silver ions found in cells in the vicinity of the nanoparticles or are they broadly distributed over the entire cytoplasm or even within the cell nucleus? These observations have to be correlated with morphological changes of cells as well as biochemical reactions in cellular media, such as changes in the intracellular distribution of Ca^2+^ during apoptosis. Such investigations require spectroscopic methods which permit high resolution imaging combined with selective probing. Imaging by conventional methods, such as optical microscopy, cannot be applied because the spatial resolution is not sufficient to probe sub-cellular details (except for novel super-resolution microscopic techniques like STED, PALM or NSOM). While such a resolution is easily provided by transmission electron microscopy (TEM), this technique requires thin samples and, hence, slicing of the samples as well as additional staining, which both might change the properties of the samples. Furthermore, energy dispersive X-ray spectroscopy (EDX) combined with TEM has only limited spectral and spatial resolution and sensitivity to provide an accurate local elemental analysis. The requirements mentioned are fulfilled, however, by scanning transmission X-ray microscopy (STXM) [72–74].

In this technique, high-brilliance, tunable synchrotron radiation in the soft X-ray regime is tightly focused, and the specimen is raster-scanned while the intensity of transmitted X-rays is recorded so that two-dimensional images are obtained. Besides a high spatial resolution (15 nm), X-ray microscopy provides a chemical contrast because of the strong variation of the absorption cross section in core level absorption. This also permits to probe a sample without the necessity to stain the local chemical environment of the absorbing site. The spectral resolution of STXM is about three orders of magnitude higher than that of EDX. Hence, chemical information of the sample with high spatial resolution is obtained. In addition, even thick (up to 10 µm) and wet samples can be studied. In spite of these advantages, the number of available setups is limited, so that STXM has only been applied to a small number of biological or biomedical samples in the past, including the investigation of local morphological changes in cells [75].

**Figure 5:**
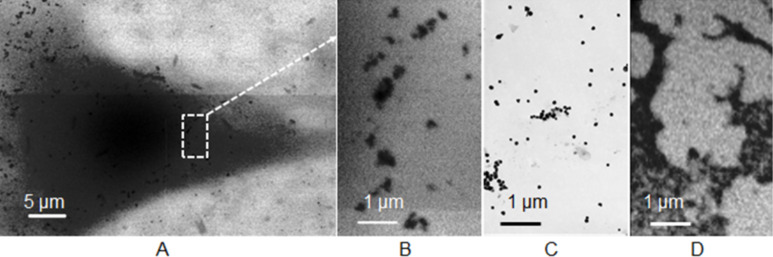
A: STXM images at 510 eV of human mesenchymal stem cells (hMSC) after 24 h of incubation with spherical silver nanoparticles (A). B: Enlarged view from image A. C: TEM image and D: STXM image at 375 eV of the same batch of silver particles before incubation.

We have investigated whether STXM can be applied to investigate the cellular uptake process of silver nanoparticles in human mesenchymal stem cells (hMSC). For this purpose, hMSC were grown on collagen-coated Si_3_N_4_-membranes and incubated for 24 h with O_2_-free aqueous dispersions of silver particles (*c* = 25 µg mL^−1^). After incubation, the loaded cells were dried and fixed [75]. Subsequently, STXM-images were recorded at the PolLux scanning transmission soft X-ray microscopy (STXM) microscope at the Swiss Light Source in the water window at 510 eV below the O K-edge, where the contrast for organic material is optimal. Imaging at the Ag M_4,5_-edges (360–390 eV) was successful for pure silver particles in the absence of any biological material (i.e., just the dried nanoparticle dispersion) on a thin (10 nm) carbon grid (Figure 5D), but failed for samples with cells. This was likely due to the insufficient contrast of the relatively low concentrated silver nanoparticles compared to the strong background signal from the biological material. Unfortunately, imaging at the Ag L_3,2_ edges (3.3–3.5 keV) with higher absorption contrast for silver was not possible at the PolLux-instrument. A strong localization of silver in the perinuclear region was observed (Figure 5A). In none of the investigated cells an indication for uptake into the nucleus was found, which is in agreement with a quantitative TEM analysis of citrate- or polyethylene glycol (PEG)-stabilized gold nanoparticles, in which no particles were identified in the nucleus [76]. Within the resolution of STXM no morphological changes of the cells were found. The particles that were taken up into the cells appear to be slightly aggregated or at least associated to larger units (see Figure 5B in comparison to particles imaged by TEM or STXM before cell incubation: Figures 5C and 5D). Probably, the particles were taken up into lysosomes and slightly aggregated therein. It is not possible to resolve individual small silver particles in cells because the spatial resolution of STXM is 15 nm.

In conclusion, the results from STXM measurements show that this method can be successfully applied to investigate uptake processes of silver nanoparticles in entire cells. However, spectro-microscopic studies are still challenging if the particle size is small and the particle concentration is low. For the imaging of the silver nanoparticle dissolution process in cells (including the localization of low concentrations of small nanoparticles as well as silver ions) imaging at the Ag L_3,2_ edges is a promising option for future work.

**Figure 6:**
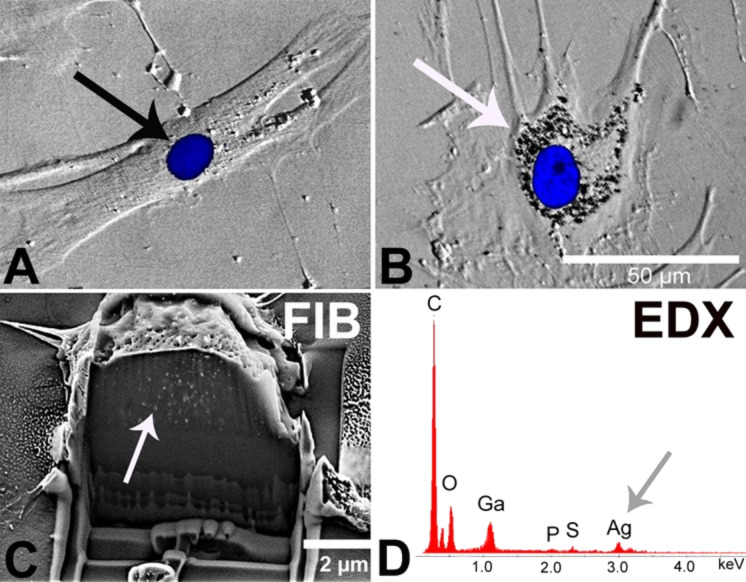
Agglomeration of internalized silver nanoparticles in hMSC analyzed by phase contrast microscopy (B), FIB/SEM (C), and EDX (D). hMSC were incubated for 24 h in the presence of 2 µg mL^−1^ silver acetate (A) as control or 20 µg mL^−1^ silver nanoparticles (B–D). Accumulated nanoparticles were detected in perinuclear areas (B, white arrow). Cell nuclei were stained with blue-fluorescent Hoechst33342 (A, black arrow; B). A single cell was cross-cut by FIB milling and the cut interface was analyzed by SEM to visualize the internalized particles (C, white arrow). The corresponding EDX spectrum of the sectioned cell shows silver signals (D, grey arrow).

Focused ion beam (FIB) and optical microscopy (phase contrast microscopy; fluorescence microscopy; confocal laser scanning microscopy) are other techniques that permit to visualize nanoparticles in cells. Therefore, hMSC were cultured in the presence of either 20 µg mL^−1^ silver (as nanoparticles) or 2 µg mL^−1^ silver ions (as silver acetate; control to separate the nanoparticle and the ion effect) at 37 °C for 24 h under cell culture conditions. This distinction was made in order to separate the effect of particles from that of ions. In the presence of silver nanoparticles, agglomerated nanoparticles were detectable in a region close to the cell nucleus (Figure 6B). In contrast, agglomerates were not observed outside the cells within the cell culture medium. Again, this is in agreement with a quantitative study performed with gold nanoparticles that showed that the agglomeration of particles occurred after uptake within 1–24 h [76]. A similar culture of hMSC in the presence of silver acetate as control did not reveal any formation of silver agglomerates (Figure 6A). In order to prove that the silver agglomerates are located inside the cells, focused ion beam milling (FIB) was applied which permits the view on cross sections of various materials by a beam of high-energy gallium ions [77–78]. After culturing hMSC in the presence of silver nanoparticles, the cells were sputtered with gold and subsequently with a tungsten layer to protect the cut surface from gallium contamination. Then the cross-sectional cutting area was analyzed by scanning electron microscopy (SEM). In different areas of silver nanoparticle-treated hMSC, silver agglomerates were detected in different areas inside individual cells (Figure 6C) in contrast to hMSC which were not exposed to silver nanoparticles (data not shown). An additional EDX-analysis confirmed the presence of silver at the intracellular agglomerate sites (Figure 6D).

Here, we demonstrated that hMSC internalize silver nanoparticles, detectable as agglomerates in the perinuclear region. Previously we have shown that in the presence of 10% fetal calf serum within the cell culture medium, the particles do not agglomerate [48]. Thus, the molecular basis of this intracellular agglomeration of silver nanoparticles is still unknown; however, as reported by Dausend et al. [79] and Harush-Frenkel et al. [80] it is likely that encapsulation in a membrane vesicle (endosome/lysosome) is involved.

Different pathways were suggested for the uptake of nanoparticles into cells such as macropinocytosis, clathrin- and caveolin-mediated endocytosis, and clathrin- and caveolin-independent endocytosis [81–83]. Other possible mechanisms such as receptor-mediated diffusion through membrane pores and passive uptake by van der Waals or steric interactions (summarized as adhesive interactions) have been suggested [84]. As we have reported, silver nanoparticles were mostly taken up by hMSC through clathrin-dependent endocytosis and macropinocytosis but not through caveolin-dependent endocytosis, as shown by flow cytometry (scattergram analysis) [77].

**Figure 7:**
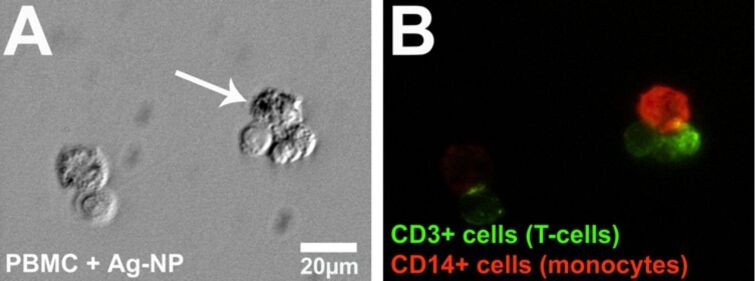
Intracellular occurrence of agglomerated silver nanoparticles in PBMC analyzed through microscopy. Representative light micrographs (phase contrast) after digital contrast enhancement (DCE filter) (A) and fluorescence micrographs (B) are shown. PBMC were treated with 15 µg mL^−1^ silver nanoparticles at 37 °C for 24 h and subsequently, the cells were labeled with specific antibodies (anti-CD3, green, and anti-CD14, red). The white arrow denotes the accumulation of particles (A) within a monocyte in the cytoplasm.

From the literature it is known that the uptake of nanoparticles is dependent on different factors such as cell type, size or functionalization of nanoparticles [81–82]. Thus, we analyzed the uptake of silver nanoparticles into human peripheral blood mononuclear cells (PBMC). These cells are host-defense cells and mainly consist of monocytes and lymphocytes (mainly T-cells). For the analysis of a cell type-specific uptake of silver nanoparticles, isolated PBMC were cultured in the presence of a subtoxic concentration of freshly prepared silver nanoparticles. After 24 h of incubation, silver agglomerates were found in monocytes (CD14+; red fluorescence) but not in the cytoplasm of T-cells (CD3+; green fluorescence) or outside the cells within the cell culture medium (Figure 7).

**Figure 8:**
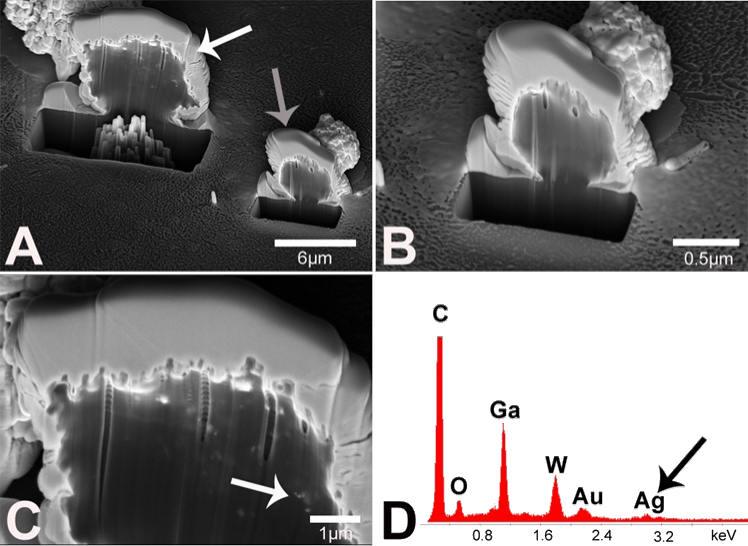
Proof of intracellular localization of silver nanoparticle agglomerates in monocytes and lymphocytes by using FIB/SEM and EDX analysis. PBMC were incubated for 24 h in the presence of 20 µg mL^−1^ silver nanoparticles, and subsequently an individual monocyte (A, white arrow) was crosscut by FIB milling. The cross section was analyzed by SEM to visualize the internalized particles. Additionally, a lymphocyte (A, grey arrow; B) within the nanoparticle-treated PBMC fraction was analyzed accordingly. The intracellular occurrence of nanoparticle agglomerates within the monocyte is denoted by a white arrow (C). No comparable signals were detected within the lymphocyte (B). The corresponding EDX spectrum of the cut monocyte (C) shows silver signals (D, black arrow).

To prove the intracellular occurrence of silver, the FIB technique was used as described above. PBMC were cultured in the presence of a subtoxic concentration of silver nanoparticles (20 µg mL^−1^) for 24 h and single monocytes or lymphocytes were crosscut with FIB for intracellular imaging. Before FIB analysis, the cells were additionally coated with a thin layer of tungsten to protect the cut area from ion contamination. Similar to the results of light microscopy (Figure 7) silver agglomerates were found in the monocytes (Figure 8C) but not in the lymphocytes (Figures 8B). As previously with hMSC, the presence of silver in the monocyte was proved by EDX (Figure 8D). The other elements C, O, W, Au and Ga are due to organic material or due to the sputtering and the FIB process. In contrast, in ion-cut lymphocytes, no silver nanoparticles were found (EDX data not shown).

In summary, direct evidence for the uptake of silver nanoparticles in hMSC and also monocytes was obtained by combined FIB/SEM experiments as well as EDX analysis [77–78], however further studies are needed to reveal the molecular uptake mechanism(s). FIB/SEM analysis represents a valuable technique to proof the nanoparticle uptake as was similarly shown by Pelka et al. [85] with HT29 cells (colon carcinoma cells) and platinum nanoparticles. Thus, FIB/SEM which presents a technique without major mechanical stress for a cell is a useful tool to detect internalized metallic nanoparticles within cells [86].

As reported in the literature, the cellular uptake of nanoparticles is a conserved process during which extracellular substances are internalized by enclosing them into vesicles called early endosomes [87]. These early endosomes mature into late endosomes after a number of steps and tend to fuse later with the acidic lysosomes [88]. Within these cell compartments, some particles may be degraded by lysosomal enzymes or they may escape from the acidic lysosomes and travel to other intracellular organelles, e.g., the Golgi complex or the endoplasmic reticulum [89].

**Figure 9:**
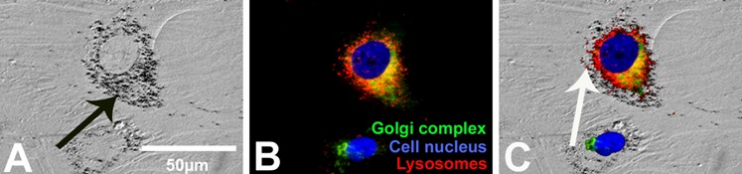
Localization of silver nanoparticles agglomerates in hMSC. A representative light micrograph after digital contrast enhancement (DCE filter; the black arrow denotes silver accumulation) (A), a fluorescence micrograph (B) and a combination of both (C) are shown. The white arrow denotes the intracellular accumulation of silver particles inside the endo-lysosomes (C). The blue fluorescence of Hoechst33342, the green fluorescence of BODIPY FL C5-ceramide and the red fluorescence of Lyso Tracker Red DND 99, were used as probes of cell nucleus, Golgi complex and endo-lysosomes, respectively.

To analyze the intracellular location of agglomerated silver nanoparticles in more detail, the Golgi apparatus, the cell nucleus and the endo-lysosomes were marked by specific fluorescent dyes. Previously, the hMSC were cultured with 20 µg mL^−1^ silver nanoparticles at 37 °C for 24 h. Phase microscopy (Figure 9A) and fluorescence microscopy (Figure 9B) images were taken of identical cell areas (merge, Figure 9C). Agglomerated silver nanoparticles were detected in perinuclear regions (Figure 9A; black arrow). As shown in Figure 9C, silver agglomerates were mainly associated with the endo-lysosomes but not with the Golgi apparatus or inside the cell nucleus.

It is known that the intracellular fate of particles depends on the size of the particles [90]. Berry et al. suggested that the uptake of nanoparticles into the cell nucleus is constrained by the pore dimension of the nucleus, because 5 nm gold nanoparticles entered the cell nucleus of human fibroblasts whereas particles larger than 30 nm were retained in the cytoplasm [91]. The silver nanoparticles used in our study have a size of 70 nm and thus, it is understandable that no silver agglomerates were found in the cell nucleus of hMSC. In addition to the particle size, the intracellular fate of nanoparticles within the cells is time- and dose-dependent [92]. As was shown by Cartiera et al. PLGA-nanoparticles were mainly found within early endosomes after 2 h of incubation in OK-cells (renal tubulus cells) but also within other compartments after a longer incubation time (4–24 h). Additionally, it is known that the endocytosis rates are specific to the cell type [93].

**Figure 10:**
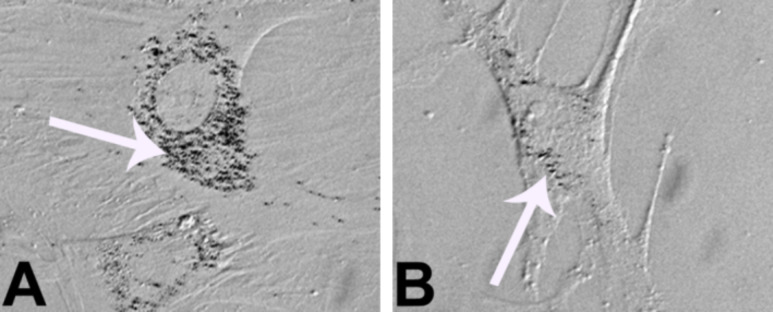
Decrease in the amount of silver agglomerates within hMSC after prolonged cell culture. hMSC were pre-incubated for 24 h with the silver nanoparticles (A), then, the cells were washed and incubated with fresh growth medium for further 72 h (B). The white arrow denotes the intracellular accumulation of silver particles.

We also demonstrated that after prolonged cell culture periods (72 h) in the absence of extracellular silver nanoparticles, the intracellular occurrence of silver agglomerates of silver-pulsed cells had decreased in a process which was clearly not related to cell proliferation under these conditions (Figure 10). Interestingly, the decrease in the number of particles was almost completely inhibited when the medium was depleted of serum (data not shown), indicating that at least the discharge of particles or ions from vesicles or other pathways at the cell surface membrane requires carrier molecules outside the cells. Interestingly, Panyam et al. have previously shown that the exocytosis of PLGA-nanoparticles in vascular smooth muscle cells was induced by the addition of BSA [94].

The understanding of the dissemination of silver nanoparticles must be related either to exoxytosis and/or to dissolution. It is complicated by the coexistence of silver in nanoparticulate and in ionic form, which will likely possess different transport characteristics. This may lead to independent or synergistic cell responses [95]. Thus, the dissemination of ingested intracellular silver nanoparticles to other cells is a critical process that obviously involves intercellular trafficking of particles followed by exocytosis and/or intracellular dissolution of silver nanoparticles to silver ions.

### Silver nanoparticles and brain cells (astrocytes)

Silver nanoparticles have been reported to damage the blood–brain barrier, to enter the brain and to cause neurotoxicity [96–98]. In addition, once nanoparticles have entered the brain, they are not efficiently cleared from the brain, in contrast to other organs, even during a long recovery period [99]. After crossing the blood–brain barrier into the brain, silver nanoparticles will immediately encounter astrocytes as these cells almost completely cover the brain capillaries with their endfeet [100]. Astrocytes are the most abundant cell type in the brain [101] and are considered to be key regulators of the homeostasis of the essential metals iron and copper in the brain [102–104]. In addition, astrocytes have the potential to accumulate toxic metals and are therefore considered to be a metal sink that protects neurons and other brain cell types against toxic metals [105]. Due to their important functions in brain metal homeostasis among the different types of brain cells, the astrocytes have recently been especially in the focus of studies on uptake and metabolism of metal-containing nanoparticles [103–104].

**Figure 11:**
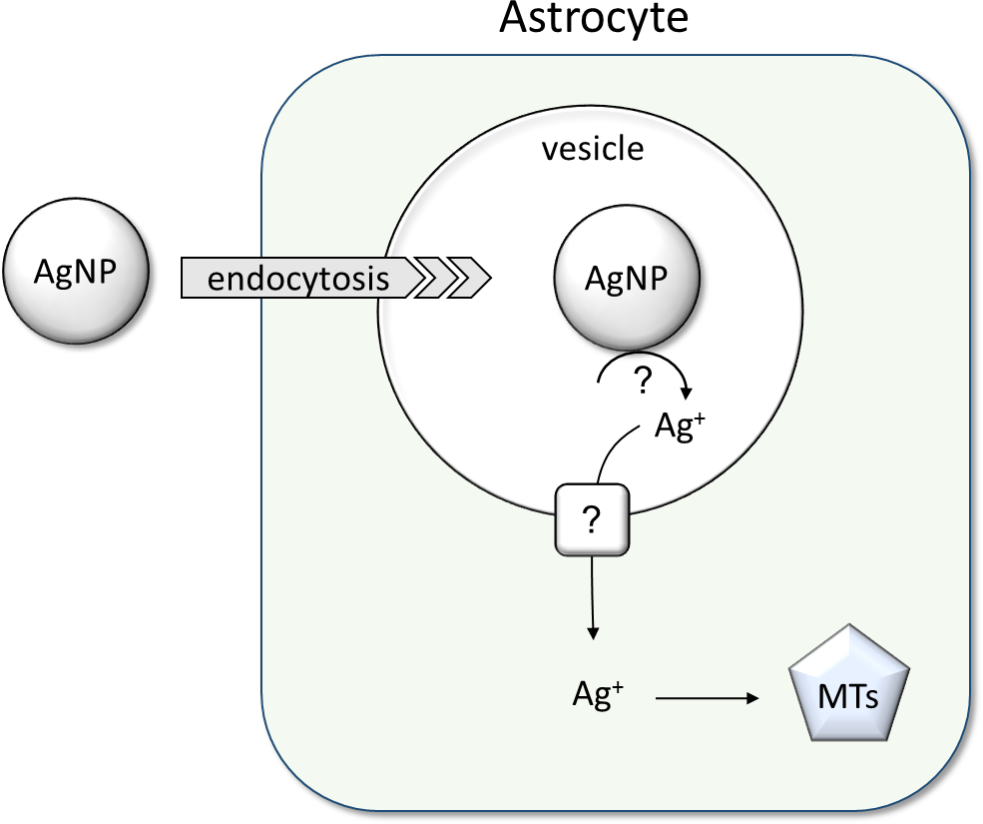
Uptake and metabolism of silver nanoparticles in brain astrocytes. Data from cultured astrocytes suggest that endocytosis contributes to the internalization of silver nanoparticles by astrocytes. Silver ions are slowly liberated from accumulated silver nanoparticles and induce the upregulation of the metal storage proteins, metallothioneins (MTs). The upregulation of these protective proteins will help to prevent severe toxicity of silver ions that are liberated from the accumulated silver nanoparticles. The mechanisms involved in the release of silver ions from internalized silver nanoparticles and in the export of such ions from the endosomes remain to be elucidated.

As model systems for brain astrocytes, primary astrocyte cultures from rat brain have frequently been used to study the consequences of an exposure of astrocytes to metal-containing nanoparticles [106–107], including silver nanoparticles [104,108–109]. The current knowledge about uptake and metabolism of silver nanoparticles in cultured astrocytes is summarized in Figure 11.

Cultured astrocytes are remarkably resistant to the potential toxicity of silver nanoparticles. Even exposure for 24 h to 100 µM (10.8 µg mL^−1^) silver as silver nanoparticles neither caused toxicity nor oxidative stress, while an incubation for 4 h with 100 µM (10.8 µg mL^−1^) silver in the form of silver nitrate strongly damaged cultured astrocytes and deprived these cells almost completely of the important antioxidant glutathione [108]. The high resistance of cultured astrocytes against silver nanoparticle-induced toxicity is consistent with the reported tolerance of astrocytes against the potential toxicity of large amounts of accumulated iron oxide nanoparticles [107], whereas astrocytes are quite vulnerable to copper oxide nanoparticles [106].

Cultured astrocytes efficiently accumulate silver nanoparticles in a process that increases their silver content proportional to the concentration of particles applied at least for incubations with silver nanoparticle dispersions containing silver concentrations of up to 300 µM (32.4 µg mL^−1^) [108]. After 4 h of incubation with 100 µM (10.8 µg mL^−1^) silver nanoparticles, cultured astrocytes contained a specific silver content of around 50 nmol (5.4 µg) per mg of protein [104,108]. Compared to an incubation at 37 °C, the specific cellular silver content is decreased to one fifth if the exposition temperature is lowered to 4 °C [108]. This indicates that the majority of silver determined in astrocytes after incubation with silver nanoparticles at 37 °C represents material that has been internalized by an active transport process through the plasma membrane. Endocytotic processes are likely to contribute to the internalization of silver nanoparticles in astrocytes as in the cell types discussed above (Figure 11), inhibitors of macropinocytosis and endosomal trafficking (chloroquine and amiloride) at least partially lower the accumulation of silver nanoparticles [108].

Accumulated silver nanoparticles appear to be quite stable in cultured astrocytes as no substantial reduction of the cellular silver content was found during a 7 d incubation of cultured astrocytes that had been loaded for 4 h with silver nanoparticles and as no delayed toxicity was found under such conditions [104]. However, some release of silver ions from internalized silver nanoparticles appears to take place in astrocytes (Figure 11), as these cells upregulate the synthesis of the metal storage proteins, i.e., metallothioneins (MTs), after exposure to PVP-coated silver nanoparticles [104]. The strong upregulation of the cellular content of these protective proteins is likely to contribute to the observed high resistance of cultured astrocytes against the potential toxicity of internalized silver nanoparticles.

The efficient accumulation of silver nanoparticles in cultured astrocytes as well as the high resistance of these cells against the potential toxicity of internalized silver nanoparticles suggest that astrocytes will also cope well in the brain with silver nanoparticles that have crossed the blood–brain barrier and further support the proposed function of astrocytes in protecting the brain against toxic metals.

### Genotoxicity of silver nanoparticles

The increasing use of silver in the form of nanoparticles raises the question whether these compounds are potentially harmful to the health of living organisms in terms of genotoxicity. Experiments were therefore also carried out with Chinese hamster fibroblasts to explore the genotoxic effects of silver nanoparticles. In mammalian cells, the toxic effects of silver nanoparticles have been explored in various cell culture systems [1]. In contrast to acute toxic effects, much less effort has been directed towards the investigation of genotoxic consequences, although these have the capacity to increase the risk of human cancer [110].

Permanent fibroblast cell lines from ovaries (CHO9, K1) and lung tissue (V79B) of Chinese hamsters are well-established in the cytogenetic analysis of potentially mutagenic triggers that induce chromosomal aberrations (CA) [111] or sister-chromatid exchanges (SCE) in mammalian cells [112]. Ionizing radiation and radiomimetic chemicals like bleomycin are potent inducers of such genome modifications, and it is generally believed that double-strand breaks (DSB) represent the central intermediate structure of such events. This model is supported by the observation that mutants that are defective in double-strand-break repair (DSB-repair) also have elevated levels of spontaneous CA and SCE and an increased sensitivity to radiomimetic drugs and ionizing radiation [113].

Although it is not yet entirely clear by which mechanisms radiomimetic drugs and ionizing radiation induce genomic DSB, there is evidence in both cases that radicals are involved [114–115]. In the case of bleomycin, reasonable models suggest that metal ions play a role during DSB formation [116]. In the case of silver nanoparticles, it has also been proposed that DNA damage may result from radicals whose formation is catalyzed from silver ions generated after uptake of silver by phagocytosis [110].

In contrast to chemical modifications of genomic DNA by reactive oxygen species (ROS) which are difficult to prove, the presence of DSB that result from a radical attack are more easily to detect. As an early response to genomic DSB, a variant of histone H2A becomes phosphorylated at amino acid 139 by members of the PI3-kinase family in the direct vicinity of the break. The phosphorylated form of H2AX (γ-H2AX) is necessary to activate the DSB-repair machinery [117] and therefore, it can be used as a tool to detect DNA-DSB by specific antibodies recognizing γ-H2AX. Staining of cells with antibodies directed against γ-H2AX results in a speckled staining of the nucleus. It is generally accepted that a single focus is representing a single DSB [118]. In the following, we describe experiments with silver nanoparticles at sub-lethal concentrations to explore the induction of CA and SCE in first metaphase chromosomes and the formation of γ-H2AX foci.

During initial experiments, we found that a concentration of 5 µg mL^−1^ silver nanoparticles added to the culture medium represented a good compromise between toxicity on the one hand and a reasonable number of chromosomal aberrations on the other hand. In preliminary lethality tests we determined the number of dead cells to less than 15% in exponentially growing CHO9 cultures (data not shown). Therefore, all further experiments were performed at a silver nanoparticle concentration of 5 µg mL^−1^. Neither a change in shape nor a detachment of cells from the dish was observed during the experiment.

**Figure 12:**
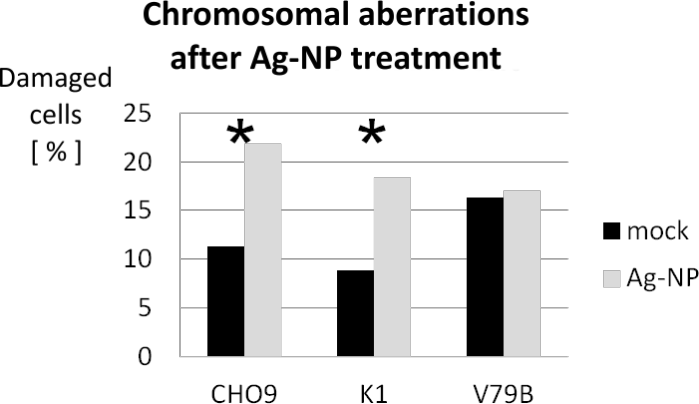
Damaged cells given in percent by scoring for CA in CHO9 (*n* = 816, *p* > 0.999), K1 (*n* = 1851, *p* > 0.999) and V79B (*n* = 726, *p* = 0.714). Black bars show data of untreated cultures and grey bars represent data observed in cultures treated with 5 µg mL^−1^ of silver nanoparticles. The asterisks indicate data that are significant when using a single sided Χ^2^ four field test. Mock = control (untreated cells).

The frequency of cells containing chromosomal aberrations (CA) was significantly increased in CHO9 and K1 cells (Figure 12). In contrast to the genotoxic effect observed in these strains, no significant increase in CA was found in the third strain V79B (Figure 12). The absence of CA in V79B is interpreted as a resistance to silver nanoparticles. Although we have not yet analyzed this phenomenon in detail, we propose that a multi-drug resistance phenomenon is most likely to be responsible for the observed results [119]. This assumption is supported by the fact that bleomycin is also unable to induce chromosome mutations in V79B (data not shown) and therefore, we can exclude an effect restricted to silver nanoparticles. In contrast, the mechanism is probably affecting multiple chemical mutagens, including silver nanoparticles.

We conclude that at the sublethal silver concentration of 5 µg mL^−1^, silver nanoparticles already have the capacity to induce genotoxic effects in Chinese hamster cells. Furthermore, in a recent study it has been shown that DNA-PKcs, which plays an important role in non-homologous-end-joining (NHEJ), is essential for the repair of DSB caused by silver nanoparticles [120]. In this study, normal human cells (IMR-90), DNA-PKcs-proficient (AA8) and DNA-PKcs-deficient (V33) Chinese hamster cell lines and human cell lines (MO59K) were exposed to silver nanoparticles. The DNA damage was greater in the normal and in the DNA-PKcs-deficient cells. Therefore this study shows the importance of DNA-PKcs in the repair of DNA-damage caused by silver nanoparticles. It also shows that a combination of silver nanoparticles and DNA-PKcs inhibition could be very interesting in the development of new anticancer therapies.

**Figure 13:**
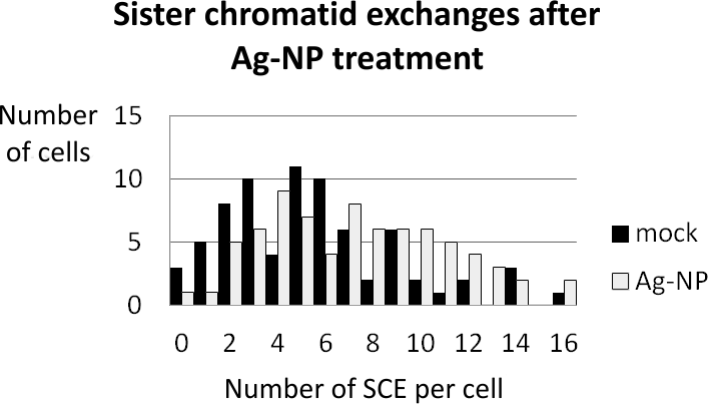
The diagram shows the distribution of sister-chromatid exchanges (SCE) in untreated cells (black bars) and silver nanoparticle treated cells (grey bars). The average number of SCE per cell increased from 5.50 in untreated cells to 7.26 in silver nanoparticle-treated cells. This shift of the average number of SCE was significant (*p* = 0.998) when using a single-sided unpaired *t*-test. In total there were 407 SCE scored in 74 untreated cells and 537 SCE in 75 silver nanoparticle-treated cells.

Since all known drugs that are mutagenic are also capable of inducing SCE, we explored levels of SCE in K1 in cells treated with silver nanoparticles and untreated cells (Figure 13) and found a significant increase in the average number of SCE in silver nanoparticle-treated cells. Since it is known that SCE and CA are both induced by DSB-forming agents, we propose that DSB play a central role in the genotoxic impact of silver nanoparticles. The formation of SCE in CHO-9 and V79B was not investigated.

**Figure 14:**
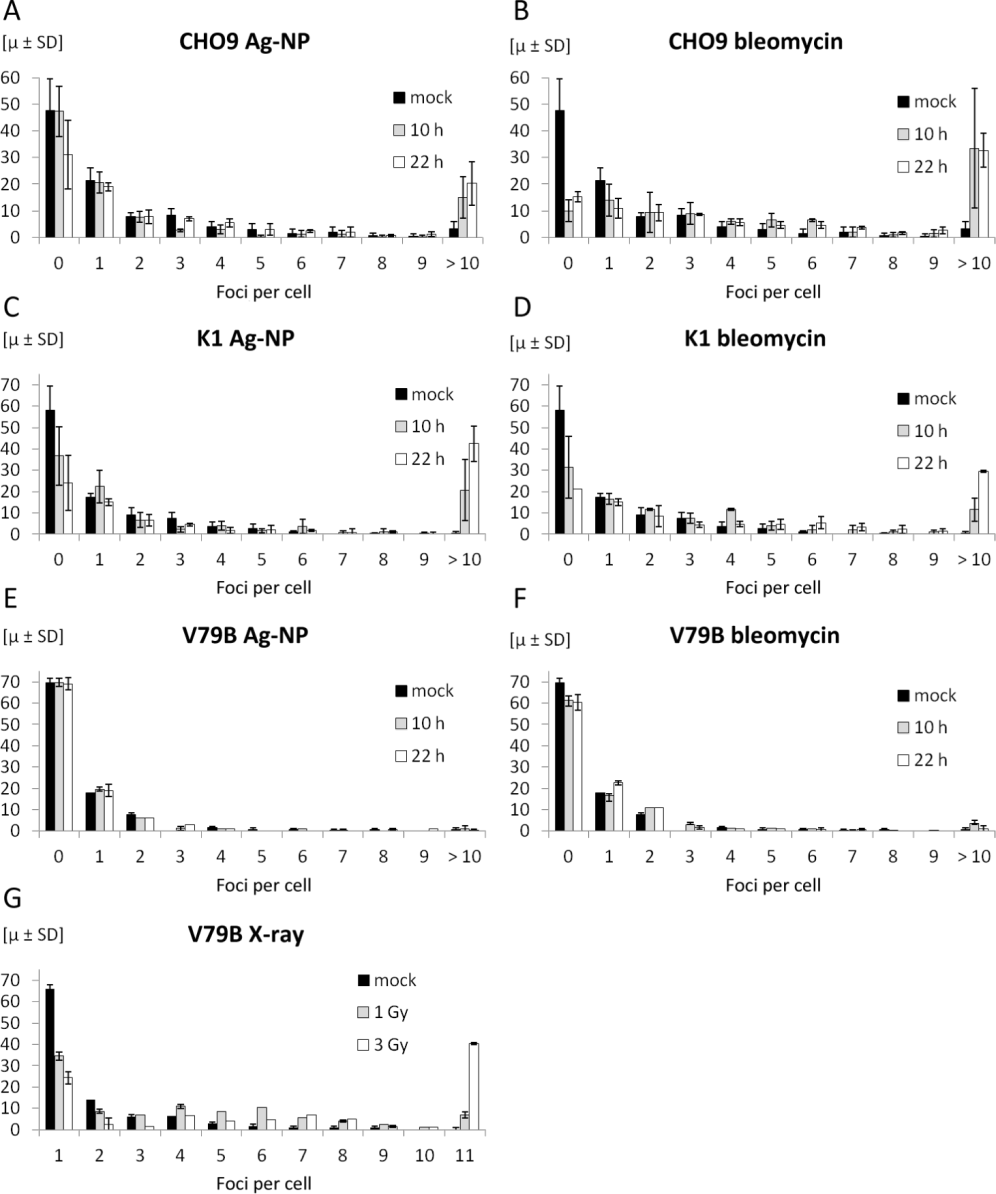
These diagrams summarize the quantification of foci formation in CHO9, K1 and V79B. Data derived from silver nanoparticle-treated cell cultures are given in Figure (A), (C), and (E) and control experiments treated with bleomycin are shown in Figure (B), (D), and (F). Panel (G) shows the proficiency of γ-H2AX foci formation in V79B cells after irradiation. These foci were not detectable in V79B cells after silver nanoparticle treatment (E) or treatment with bleomycin (F).

Confluent silver nanoparticle-treated cells have been stained after 10 h and 22 h with a monoclonal antibody recognizing γ-H2AX. The quantifications of the number of foci induced in the three cell lines CHO9, K1 and V79B are given in Figure 14. About 100 nuclei were scored at each time point, and the number of foci in each nucleus was counted. The cells were treated with either silver nanoparticles (Figure 14A, 14C, 14E) or with bleomycin (Figure 14B, 14D, 14F). The distribution of foci formation is shown in each figure. Cells with more than ten foci are rare in untreated cells but this fraction is significantly increased in CHO9 and K1 cells which were treated with either silver nanoparticles or bleomycin for 10 h, respectively. To our surprise, this pattern did not hold for the third cell line V79B, neither for silver nanoparticles nor for bleomycin. We conclude that DSB are not formed in V79B and that this cell line is resistant against mutagenesis caused by silver nanoparticles and bleomycin at the concentration levels applied during this study. This finding also confirms the coincidence of the lack in DSB formation with the absence of CA in V79B. To test this hypothesis and to exclude any technical problems (e.g., with the antibody used), we induced γ-H2AX by using X-rays instead of radiomimetic treatment or silver nanoparticle exposure (Figure 14G). Untreated cells (black bars) and cells treated with 1 Gy (grey bars) and 3 Gy (open bars) were collected and fixed 30 min after irradiation and stained for γ-H2AX foci. As shown in Figure 14G, γ-H2AX foci have been detected also in V79B cells after irradiation. Therefore, we can exclude technical reasons for the deficiency of γ-H2AX foci in V79B cells following treatment with silver nanoparticles or bleomycin. Last but not least, multidrug resistance has been reported earlier for the V79 cell line, which is a related cell line to V79B used during this study [121]. It is not clear whether the resistance found in V79B compared to the other two cell lines CHO9 and K1 is related to the origin of the cell line or whether V79B has acquired this phenotype during cell culture processing. While V79B was originally isolated from lung tissue, the two other cell lines used in our study were derived from ovaries. The observed data raise the question whether tissues have a different sensitivity towards silver nanoparticles or possibly against free radicals.

In CHO K1 cells treated with silver nanoparticles, a significant number of cells with endoreplicated (ER) chromosomes was found. ER chromosomes result from aberrant cell cycles by two rounds of replication without chromatid separation in between. Although cells with ER chromosomes were already detectable during regular experiments with cell cultivation times of 22 h, the number of those cells could be further increased by extending the cultivation time to 40 h. Under these conditions, 10 to 20% of all metaphases contained ER chromosomes.

**Figure 15:**
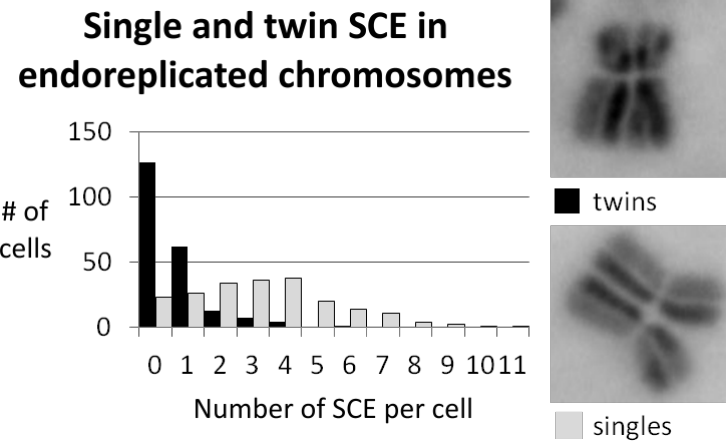
The diagram shows the distribution of twin SCE (black bars) and single SCE (grey bars) in CHO K1 cells, treated with silver nanoparticles. The average number of SCE per cell changes from µ = 0.612 for twin SCE (*n* = 214) to 3.115 for single SCE (*n* = 208; *p* > 0.999). Images on the right side show examples for twin SCE and single SCE.

We do not yet know the reason for this phenomenon so far, but we used this as a tool to explore SCE frequencies taking place during the first (twins) and the second round of replication (singles), following differential staining of the sister chromatids. The distribution of single SCE and twin SCE is shown in Figure 15. It turned out that there was a significant increase in single SCE compared to twin SCE. We assume that the difference in the number of single SCE and twin SCE results from different frequencies of SCE formation during the first and second round of replication and is a consequence of the genotoxic effect caused by the silver nanoparticles.

This is possible because SCE events from the first replication result in twin SCE, while in the second round of replication, SCE result in single SCE. To our surprise, the frequency of single SCE was strongly increased compared to the frequency of twin SCE. We explain this by an increase of the mutagen concentration inside the cell, which is controlled by a rate-limiting and relatively slow process. Therefore, the concentration of mutagen increases inside the cell between the first and second round of replication.

We propose that there are two possibilities for the nature of the rate-limiting component. Firstly, the uptake mechanism might be slow. This would be consistent with the model that silver nanoparticles are transported into the cell by endocytosis. Alternatively, the dissolution of silver nanoparticles might be rate-limiting due to the moderate stability of the particles at least outside the cell. Last but not least, a combination of both effects also cannot be excluded. Further experiments are necessary to understand this phenomenon in more detail.

### The lung and silver nanoparticles

The wide variety of silver nanoparticles applications allows for different routes of entry into the human body [122]. Silver nanoparticle exposures to humans can occur orally, e.g., by colloidal solutions, dermally, e.g., in contact with jewelry or burn creams, and by inhalation of dusts or fumes. The latter is a major concern in occupational settings when handling silver nanoparticles at the point of production and the manufacture of nanoparticle-containing materials. However, since the antibacterial properties of silver nanoparticles and silver salts promote an increased use in personal care products, aerosolized silver nanoparticles and silver salts in spray products such as deodorants or pharmaceuticals are therefore of particular significance to the lung [1,12–13,123].

In vivo studies using animals or humans provide the benefit of having a complete organism displaying the full range of biological responses to a given treatment. Human case studies on silver nanoparticles are only performed to reveal the side effects of an unintended exposure to silver, misuse of colloidal silver solutions, or work-related exposure and not to investigate the toxic mechanisms of newly developed materials. A recently published animal study suggests that exposure of rats to silver nanoparticles (50 and 250 µg) by intratracheal instillation can cause moderate pulmonary toxicity in vivo, but only at rather high concentrations [124].

Ex vivo approaches, such as isolated-perfused lungs or precision-cut lung slices (PCLS), have been developed as an alternative to in vivo studies [125]. They allow for a more detailed view on the direct interaction of nanoparticles with whole organs or parts of them. Moreover, they can contribute to the reduction of animal experiments and also have a better cost and benefit ratio, as in the case of PCLS one organ can be used for several exposure conditions. The exposure of cultured rat PCLS to silver nanoparticles (10, 20 and 30 µg mL^−1^) under submerged conditions for 4 and 24 h resulted in only weak cytotoxicity (LDH release), but did not induce a proinflammatory response (CXCL-1 and TNF-α release). Interestingly, multiphoton microscopy revealed that the silver nanoparticles were localized predominantly at the cut surface but not inside PCLS, indicating that the particles did not reach the inner PCLS tissue regions [126].

**Figure 16:**
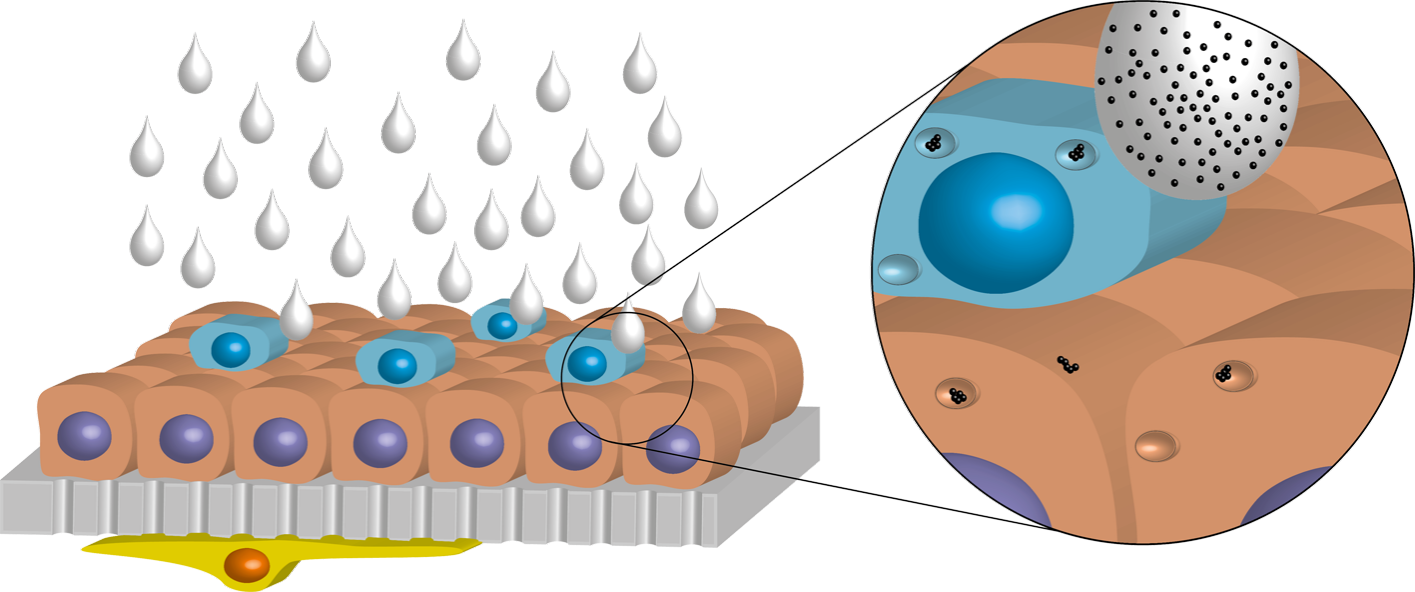
Schematic image of the triple-cell co-culture model consisting of MDMs (blue), A549 cells (red), a porous membrane (grey), and dendritic cells (yellow). The cells were cultured at the air-liquid interface for exposure of silver nanoparticle suspensions by nebulization. Reproduced from [125].

However, ex vivo approaches are complex and difficult to control in a standardized laboratory environment, therefore sample administration might not reflect the original conditions anymore. Furthermore, they experience similar disadvantages as in vivo experiments due to the fact that the source is still an animal. Following the aforementioned considerations, another approach to assess possible risks of silver nanoparticles by inhalation was to use a cellular model of the human airway/alveolar epithelial barrier. The human 3D cell culture model consists of three cell types, i.e., alveolar epithelial cells, macrophages, and dendritic cells as described in [127]. To imitate the lung organ structure, an A549 cell layer was cultured on porous membranes with human monocyte-derived macrophages (MDMs) on top on the apical side and monocyte-derived dendritic cells (MDDCs) underneath on the basal side. This 3D co-culture system was further cultivated at the air–liquid interface to approximate the in vivo situation in the lungs [128]. Although essential parts are missing like the lymph and blood circulation, muscles, connective tissue, or the respiration movement, it permits to investigate the interaction of silver nanoparticles with lung cells at a higher complexity through an in vitro method without using invasive animal model tests. To make use of the realistic culture conditions, a specifically designed exposure system was employed which allows the nebulization of a defined nanoparticle suspension onto the cells at the air–liquid interface (ALI), mimicking the inhalation of nanoparticles (Figure 16).

This air–liquid interface cell exposure (ALICE) system has been intensively characterized [129] and allows for exact measurements of the deposited amount of nanoparticles revealing direct dose–response relations, which can be more difficult to achieve through in vivo experiments. In a previous study with 20 nm citrate-coated silver nanoparticles, no effects were observed in cultures exposed to 0.03 and 0.3 µg silver cm^−2^ [130].

In a further study, PVP-coated silver nanoparticles were used with the same cell and exposure system [125]. The cells were exposed to three different concentrations of silver nanoparticles resulting in a final deposition of 0.03, 0.3, and 3 µg silver cm^−2^. A transient increase of mRNA expression encoding tumor-necrosis factor-alpha (TNF-α) could be detected after 4 h only for the highest silver nanoparticle concentration. The protein levels of TNF-α were, however, not increased after 4 and 24 h, and moreover, no other effects, i.e., oxidative stress and different pro-inflammatory cytokines/chemokines, were observed. Compared to a representative lung deposition attributable to occupational exposure of 5–100 nm silver nanoparticles calculated after 24 h, as described by Gangwal et al. [131], an expected deposited dose in elevated atmospheric concentrations of 1 mg silver m^−3^ varies between 0.061–0.15 µg silver cm^−2^. Consequently, the highest dose applied to cells at ALI in this study (i.e., 3 µg silver cm^−2^) suggests to be more accurate, according to [131], a working lifetime or higher concentration. Concluding these in vitro experiments, representative silver nanoparticle concentrations are not expected to induce acute cytotoxic or pro-inflammatory effects after 24 h exposure in a realistic environmental setting, therefore implicating to have a low impact on the human health. However, bioaccumulation of silver nanoparticles over time may induce secondary effects, which cannot be ruled out by such in vitro studies. Chronic exposure and inhalation studies need to address this issue in the future.

As summarized in Table 2, the effects induced by the PVP-coated silver nanoparticles in three different lung systems, i.e., a 3D lung model composed of human cells, PCLS from rat, and intratracheal instillation to rats, are difficult to directly compare and interpret. Several reasons can explain the observed differences, such as the different species used (human, rat) as well as differences in cell numbers and types. In addition, the exposures were also different and carried out once at the air–liquid interface, once in submerged conditions, and once through instillation. In the latter two methods, the determination of the exact dose deposited per area is not possible. Nevertheless, one observation is similar, namely that effects, such as cytotoxicity and/or (pro-)inflammatory responses are only induced by higher concentrations of silver nanoparticles.

**Table 2:** Comparison of the cytotoxic and (pro-)inflammatory responses induced in different model systems exposed to PVP-coated silver nanoparticles.

model		species	dose (silver)	cytotoxic response	(pro)-inflammatory response

in vitro	epithelial airway barrier model, cultured at the air-liquid interface	human	0.03 µg cm^−2^ 0.3 µg cm^−2^ 3 µg cm^−2^	none none none	none none transient^a^
ex vivo	precision-cut lung slices (PCLS)	rat	10 µg mL^−1^ 20 µg mL^−1^ 30 µg mL^−1^	none weak weak	none none none^b^
in vivo	intratracheal instillation	rat	50 µg 250 µg	none moderate	none moderate^c^

^a^Significant increase in mRNA for TNF-α after 4 h, which decreased to control values after 24 h. No increase in TNF-α protein after 4 h and 24 h as determined by ELISA [125]. ^b^Significant, but weak increase in LDH release after incubation for 4 h and 24 h. No increase in CXCL-1 or TNF-α protein as determined by ELISA [126]. ^c^Significant increases in BALF LDH, total protein, and cytokine levels as well as in BALF neutrophil numbers 24 h after intratracheal instillation of 250 µg silver nanoparticles [124].

### Interaction of silver nanoparticles with the human skin barrier and keratinocytes

Silver is widely used in dermatology and health care as antibacterial and anti-inflammatory agent [132]. Especially wound dressings and gels containing nanoscale silver particles are an achievement of biomedical engineering to prevent and handle wound infections. Silver nanoparticles slowly and continuously release silver ions, thus providing a sustained antibacterial effect [1,133]. Especially when applied to inflamed skin and wounds, however, the topically applied particles also have easy access to living skin cells and may easily enter the systemic circulation [134–135]. For a proper risk assessment and in order to improve the therapeutic window of such antimicrobial materials, possible cytotoxic effects need to be carefully distinguished. For instance, Foldbjerg et al. showed in studies with 70 nm sized silver nanoparticles and silver nitrate on THP-1 monocytes that Ag^+^ ions from silver nitrate were much more toxic than the particles although a particle-related toxicity was not studied [136]. We have investigated the interaction of silver nanoparticles (10–50 µg mL^−1^) on porcine ear skin samples and on the human keratinocyte cell line HaCaT. The interaction of silver nanoparticles with the skin barrier (i.e., the stratum corneum) was investigated by confocal Raman microscopy. The mean measured penetration depth in intact skin was 4.4 ± 1.5 µm, which is in accordance with other investigations on solid particles in this size range showing that solid particles remain, to a large extent, in the superficial stratum corneum [137]. However, even in intact skin, when measurements were carried out by exploiting the surface-enhanced Raman scattering (SERS) effect which is typical for silver nanoparticles in this size range, a SERS signal was detected down to a depth of 10–18 µm [138].

Especially with regard to repetitive applications on inflamed skin and wounds, direct silver nanoparticle exposure of vital epidermis cells and cellular uptake is very likely to occur. We therefore investigated possible biological effects of such a cellular uptake in in vitro experiments on silver nanoparticle-treated HaCaT cells by transmission electron microscopy and XTT viability assay. We showed that a decrease of the cell viability depended on the silver nanoparticle concentration and the percentage of fetal calf serum in cell incubation media [139]. When toxic concentrations of silver nanoparticles were applied to HaCaT cells (nanoparticles stored for a long time under oxygen atmosphere) a degeneration of the cell nuclei and the cell membranes was observed, leading to cell death [139]. Concentration-dependent effects of 80 nm-sized nanoparticles on human epidermal keratinocytes have also been studied by Samberg et al. [140]. Similar results were published by Miura et al., who compared the toxicity of silver nanoparticles to that of silver nitrate on HeLa cells [141].

**Figure 17:**
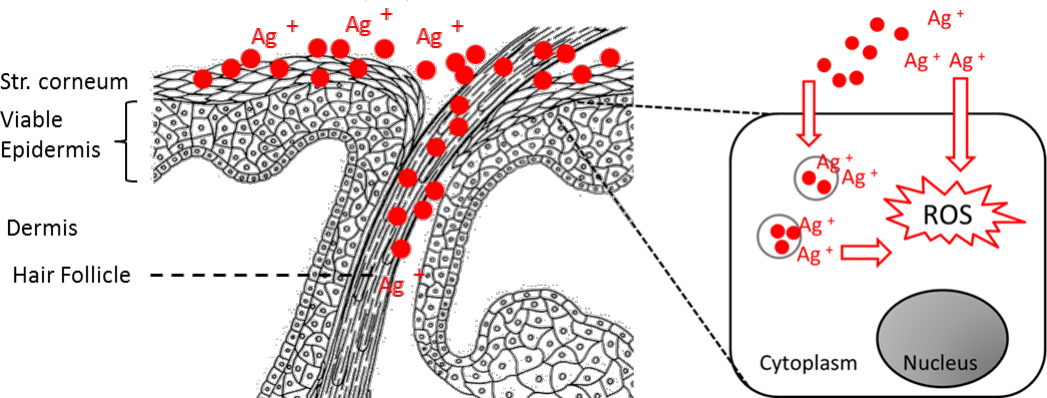
Cytotoxic effects and free radical production by silver nanoparticles per se versus the effects due to secondary Ag^+^ ion release.

Because the main mechanism of silver nanoparticles cytotoxicity was reported to be related to free radical species produced by the interaction of silver ions with the cell component, we used EPR spectroscopy to detect the silver nanoparticle-mediated intracellular production of reactive oxygen species (ROS). To differentiate between the effects of silver ions released during particle storage and those of silver ions released after particle intracellular uptake, we investigated two different silver nanoparticle dispersions: One produced and stored under air (O_2_), with a comparatively high silver ion (Ag^+^) concentration due to oxidative dissolution, and one produced and stored under argon (Ar) with a low silver ion content [20]. One hour after incubation with the cells, significant differences were found between the two silver nanoparticle samples at the different concentrations used [139]. Particles produced and stored under air induced ROS to a significantly higher extent than silver nanoparticle stored under argon. These findings indicate that the silver ions released during particle storage are responsible for most of the intracellular radicals induced by the investigated silver nanoparticle dispersions and confirm the role of silver ions in silver nanoparticle-mediated cytotoxicity (Figure 17). Hence, despite the substantial value of silver particle-containing formulations for skin surface antisepsis, the cytotoxicity of silver nanoparticles towards skin cells is detrimental to their broad use in wound treatment. This needs to be carefully considered especially when repetitive applications on skin with a disrupted barrier are required over longer period of times. Storage of silver nanoparticle formulations under inert atmosphere might be an option to avoid side effects due to high concentrations of silver ions and to take advantage of the slow-release properties of particulate silver.

Topically applied solid particles largely remain in the superficial horny layers of skin which provides a strong rationale for the use of silver nanoparticles in skin surface antisepsis. However, skin barrier translocation at natural interruptions such as hair follicles has been reported [134–135], and especially when applied on inflamed skin and wounds, contact to viable epidermis and cellular uptake inevitably occur.

## Conclusion

The results from all studies are summarized in Table 3.

**Table 3:** Summary of all results obtained for PVP-coated silver nanoparticles.

investigated property	results	reference

physical properties	diameter of the metallic core of 70 nm; hydrodynamic diameter of 120 nm; zeta potential of −20 mV	[33]
dispersability	dispersable in water; agglomeration in salt-containing solutions; good dispersability in the presence of proteins	[48]
dissolution	dissolves in the presence of oxygen under the release of silver ions	[20,33]
protein adsorption	enclosed by a protein corona in biological media	[55,69]
uptake by cells	endocytosis (hMSC, primary T-cells, primary monocytes, astrocytes)	[77–78,108]
visualization inside cells	fluorescence microscopy; X-ray microscopy; FIB/SEM	[77–78]; this work
cytotoxicity (particles prepared and stored under air)	hMSC: 25–50 µg mL^−1^; primary T-cells: >50 µg mL^−1^; primary monocytes: 50 µg mL^−1^; astrocytes: no toxicity after 24 h exposure to 10 µg mL^−1^ and after 4 h incubation with up to 30 µg mL^−1^ for 4 h; HaCaT cells: ≥30 µg mL^−1^	[33,48,108–109,139,142]
bactericidal effect	*E. coli*: 12.5 to 50 µg mL^−1^; *S. aureus*: 20–50 µg mL^−1^	[142]
genotoxicity	induces the formation of DNA double strand breaks (DSB), chromosomal aberrations and sister-chromatid exchanges (CHO9, K1, V79B cell lines) at 5 µg mL^−1^	this work
intratracheal instillation of rats	moderate pulmonary toxicity (250 µg Ag)	[124]
precision-cut lung slices (PCLS) (rat)	moderate pulmonary toxicity (30 µg Ag mL^−1^)	[126]
epithelial airway barrier model, cultured at the air–liquid interface	transient increase in mRNA for TNF-α after 4 h for 3 µg Ag cm^−2^	[125]
skin exposure	penetration to 10–18 µm on porcine ear skin 16 h after application of 12 µg Ag cm^−2^	[138]

As demonstrated above, we conclude that well-defined and well-characterized nanoparticles are necessary in targeted investigations to permit a comparison of the results from different laboratories.

Within the Priority Program SPP1313 of the Deutsche Forschungsgemeinschaft (DFG) we had the opportunity to use thoroughly characterized PVP-capped silver nanoparticles that were stored and characterized under well-defined conditions. This particle type was then distributed to different laboratories to test the possible interaction of the particles with various biological systems, i.e., single cell cultures (mesenchymal cells, astrocytes, keratinocytes, fibroblasts), more complex cell models (3D model of the human epithelial airway wall, PCLS, porcine ear skin samples), or in animals (rats). Although the same silver nanoparticles were used, quite different responses in the biological systems were observed. This can be explained by the use of different cell species, the different endpoints that were assessed, and also the different exposure routes of the particles.

In general, it can be said that only higher concentrations of silver nanoparticles induced biological responses in single cell cultures, in more complex systems and in animal experiments. Only for the fibroblast cultures, a genotoxicity was observed at rather low concentrations. Further coordinated efforts will be needed to explore the possible effects that silver nanoparticles can induce in (human) cells.

## Experimental

### Synthesis of silver nanoparticles [20]

PVP-coated silver nanoparticles were synthesized by reduction with glucose in the presence of PVP according to Wang et al. [28]. Briefly, 2 g glucose and 1 g PVP were dissolved in 40 g water and heated to 90 °C. Then 0.5 g AgNO_3_ dissolved in 1 mL water were quickly added. The dispersion was kept at 90 °C for 1 h and then let to cool to room temperature. The particles were collected by ultracentrifugation (29,400*g*; 30 min), redispersed in pure water and collected again by ultracentrifugation. Thereby NO_3_^−^, excess glucose and its oxidation products, excess PVP, and excess Ag^+^ were removed. The silver nanoparticles were then redispersed in water by ultrasonication. The final silver concentration in all dispersions was determined by atomic absorption spectroscopy (AAS). Poly(*N*-vinylpyrrolidone) (PVP K30, Povidon 30; Fluka, molecular weight 40,000 g mol^−1^), silver nitrate (Roth, p.a), D-(+)-glucose (Fluka, p.a.) were used.

Scanning electron microscopy (SEM) was performed with a FEI Quanta 400 ESEM instrument in high vacuum without sputtering. The 10 µL of dispersion was dripped on silicon wafer and dried at room temperature in air. The hydrodynamic diameter and the zeta potential of the nanoparticles were measured through dynamic light scattering (DLS) by using a Malvern ZetasizerNano ZS. The polydispersity index (PDI) was below 0.3 in all cases. The concentration of silver was determined by atomic absorption spectroscopy (AAS; Thermo Electron Corporation, M-Series). The detection limit was 1 µg L^−1^. Dialysis experiments were carried out as described earlier [20,33].

### Circular dichroism spectroscopy

Experimental details of the circular dichroism (CD) experiments were described earlier [54,63,69]. Briefly, measurements were carried out with an AVIV 62 A DS CD spectrometer with a slit width of 1 µm and a scanning step size of 0.1 nm. Measurements were performed in a 1 mm path length Suprasil^®^ quartz cuvette which was carefully cleaned between the individual measurements with Hellmanex^®^ (Hellma) cuvette cleaning agent and subsequently with deionized water. Protein concentrations were kept constant throughout the measurements at 0.52 or 0.052 mg mL^−1^ and the total volume of the combined protein/nanoparticle solution was also kept constant by adding deionized water. Nanoparticles and proteins were incubated for 3 h at room temperature prior to the measurements to ensure the formation of an "equilibrated" protein corona. All nanoparticles remained stable in solution on the timescale of our experiments with no sign of agglomeration.

### Scanning transmission X-ray microscopy

25 µg mL^−1^ of O_2_-free aqueous dispersions with silver nanoparticles were incubated for 24 h with human mesenchymal stem cells (hMSC) and HaCaT cells (human keratinocytes) grown on collagen-coated Si_3_N_4_-membranes [75]. After incubation the cells were dried and fixed [75]. The measurements were performed at the PolLux scanning transmission soft X-ray microscopy (STXM) microscope at the Swiss Light Source. Details on the microscope and the beam line are given in [143] and [137]. Images were recorded at selected photon energies between 270 and 550 eV. Image processing was carried out by using the aXis2000 software (http://unicorn.mcmaster.ca/aXis2000.html).

### Biological studies

Human mesenchymal stem cells were cultured and incubated as reported earlier [77–78]. Astrocytes were cultured and incubated as reported earlier [108–109]. For genotoxicity assays, we used three different permanent Chinese hamster cell lines (CHO9, CHOK1 V79B). Cells were cultivated in McCoy’s 5A Medium supplemented with 10% fetal calf serum, penicillin (100 units mL^−1^), streptomycin (100 µg mL^−1^), and L-glutamin (7.5 mL L^−1^). Cells, cell culture conditions and the preparation of metaphase chromosomes were described earlier [144–145]. Metaphase plates scored for sister-chromatid exchanges are stained by using the Fluorescence-plus-Giemsa (FPG) protocol. Briefly, the slides from cells grown in 20 Mol-6 for two rounds of DNA replication were incubated for 8 min in Hoechst 33258 (4.5 mg mL^−1^ in PBS) followed by UV (260 nm) irradiation at 60 °C and finally staining with Giemsa (5%). All other metaphases were uniformly stained with Giemsa. A BX-60 fluorescence microscope (Olympus Optical Co, Japan) was used for scoring CA and SCE from metaphase spreads. Double-strand breaks were induced either by addition of bleomycin to cultured cells for 12 h at a final concentration of 100 µg mL^−1^ or by irradiation with X-rays at 1 Gy. Detection with antibodies was performed as described [145] using anti-γ-H2AX primary antibody (1:400) (Millipore, USA) and Alexa-Fluor®594-conjugated goat anti-mouse secondary antibody (1:2000) (Invitrogen, Darmstadt). The cells were mounted in Vectashield (Invitrogen, Darmstadt).

Details on the in vivo intratracheal rat installation experiments are given in [124]. Details on the experiments with precision-cut lung slices (PCLS) are given in [126]. Details on the triple-cell 3D culture are described in [125,127–129]. Details on the interaction of silver nanoparticles with the human skin barrier and keratinocytes are described in [135,137,139].

## Authors

Sebastian Ahlberg^1^, Alexandra Antonopulos^2^, Jörg Diendorf^3^, Ralf Dringen^4^, Matthias Epple^3*^, Rebekka Flöck^2^, Wolfgang Goedecke^2^, Christina Graf^5^, Nadine Haberl^6^, Jens Helmlinger^3^, Fabian Herzog^7^, Frederike Heuer^2^, Stephanie Hirn^6^, Christian Johannes^2^, Stefanie Kittler^3^, Manfred Köller^8^, Katrin Korn^2^, Wolfgang G. Kreyling^9^, Fritz Krombach^6^, Jürgen Lademann^1^, Kateryna Loza^3^, Eva M. Luther^4,10^, Marcelina Malissek^11^, Martina C. Meinke^1^, Daniel Nordmeyer^5^, Anne Pailliart^2^, Jörg Raabe^12^, Fiorenza Rancan^1^, Barbara Rothen-Rutishauser^7^, Eckart Rühl^5^, Carsten Schleh^13^, Andreas Seibel^2^, Christina Sengstock^8^, Lennart Treuel^11,14^, Annika Vogt^1^, Katrin Weber^2^ and Reinhard Zellner^11^

## Addresses

^1^Department of Dermatology and Allergy, Charité-Universitätsmedizin Berlin, Berlin, Germany, ^2^Genetics and Center for Medical Biotechnology (ZMB), University of Duisburg-Essen, Universitaetsstr. 5–7, 45117 Essen, Germany, ^3^Inorganic Chemistry and Center for Nanointegration Duisburg-Essen (CeNIDE), University of Duisburg-Essen, Universitaetsstr. 5–7, 45117 Essen, Germany, ^4^Center for Biomolecular Interactions Bremen and Center for Environmental Research and Sustainable Technology, Faculty 2 (Biology/Chemistry), University of Bremen, PO. Box 330440, 28334 Bremen, Germany, ^5^Free University of Berlin, Physical and Theoretical Chemistry, Takustr. 3, 14195 Berlin, Germany, ^6^Walter Brendel Centre of Experimental Medicine, Ludwig-Maximilians-Universität München, 81377 Munich, Germany, ^7^Adolphe Merkle Institute, Université de Fribourg, 1723 Marly, Switzerland, ^8^Bergmannsheil University Hospital/Surgical Research, Ruhr-University Bochum, Bürkle-de-la-Camp-Platz 1, 44789 Bochum, Germany, ^9^Institute of Epidemiology 2 and Institute of Lung Biology and Disease, Helmholtz Center Munich, 85764 Neuherberg/Munich, Germany, ^10^nanoTherics Ltd, Innovation Centre 4, Keele University Science and Business Park, Keele, Staffordshire, ST5 5NL, United Kingdom, ^11^Physical Chemistry, University of Duisburg-Essen, Universitaetsstr. 5–7, 45117 Essen, Germany, ^12^Swiss Light Source, Paul Scherrer Institut (PSI), 5232 Villigen, Switzerland, ^13^Abteilung Gesundheitsschutz, Berufsgenossenschaft Holz und Metall, 81241 Munich, Germany and ^14^Fraunhofer ICT-IMM, Carl-Zeiss-Str. 18–20, 55219 Mainz, Germany
